# Pharmacological rescue of the G85E CFTR variant by preclinical and approved modulators

**DOI:** 10.3389/fphar.2024.1494327

**Published:** 2024-11-18

**Authors:** Valeria Tomati, Valeria Capurro, Emanuela Pesce, Cristina Pastorino, Elvira Sondo, Mariateresa Lena, Anna Borrelli, Federico Cresta, Stefano Pantano, Francesca Collini, Pietro Ripani, Vito Terlizzi, Cristina Fevola, Stefano Costa, Maria Cristina Lucanto, Federico Zara, Tiziano Bandiera, Renata Bocciardi, Carlo Castellani, Luis J. V. Galietta, Nicoletta Pedemonte

**Affiliations:** ^1^ UOC Genetica Medica, IRCCS Istituto Giannina Gaslini, Genova, Italy; ^2^ Department of Neurosciences, Rehabilitation, Ophthalmology, Genetics, Maternal and Child Health (DINOGMI), University of Genoa, Genova, Italy; ^3^ Telethon Institute of Genetics and Medicine (TIGEM), Pozzuoli, Italy; ^4^ UOSD Centro Fibrosi Cistica, IRCCS Istituto Giannina Gaslini, Genova, Italy; ^5^ UOSD CRR Fibrosi Cistica, P.O. San Liberatore, Atri, Italy; ^6^ Department of Pediatric Medicine, Meyer Children’s Hospital IRCCS, Cystic Fibrosis Regional Reference Center, Florence, Italy; ^7^ Centro Hub Fibrosi Cistica, Azienda Ospedaliera Universitaria Policlinico G. Martino, Messina, Italy; ^8^ D3-PharmaChemistry, Fondazione Istituto Italiano di Tecnologia, Genova, Italy

**Keywords:** CFTR, correctors, gating, theratyping, nasal, modulators

## Abstract

**Introduction:**

Cystic Fibrosis (CF) is a genetic disease due to loss-of-function mutations of the CFTR channel. F508del is the most frequent mutation (70% of alleles in Italy), while other mutations have much lower frequency. Among them, G85E (0.4% frequency globally, 1.13% in Italy) emerges as a mutation characterized by a severe CFTR folding and trafficking defect.

**Methods:**

To investigate the pharmacological responsiveness of the G85E-CFTR variant, we performed a functional and biochemical characterization in heterologous expression systems and *ex vivo* models based on patient-derived human nasal epithelial cells (HNEC).

**Results:**

Our study demonstrated that treatment of primary airway cells with elexacaftor and tezacaftor causes a significant (although modest) rescue of CFTR function, that reaches 15%–25% of the activity measured in non-CF epithelia. A detrimental effect of chronic treatment with ivacaftor, further limiting G85E rescue, was also observed. A higher rescue of CFTR function, up to 25%–35% of the normal CFTR activity, with no evidence of negative effects upon chronic potentiator treatment, can be achieved by combining elexacaftor with ARN23765, a novel type 1 corrector endowed with very high potency. Importantly, dose-response relationships suggest that G85E might alter the binding of type 1 correctors, possibly affecting their affinity for the target.

**Discussion:**

In conclusion, our studies suggest that novel combinations of modulators, endowed with higher efficacy leading to increased rescue of G85E-CFTR, are needed to improve the clinical benefit in patients for this variant.

## 1 Introduction

Cystic fibrosis (CF), one of the most frequent genetic diseases, is caused by loss-of-function variants in the *CFTR* gene. The resulting CFTR mutant proteins are partially or totally unable to perform their normal function to transport Cl^−^ across the plasma membrane of many types of cells, particularly of epithelial type (Cutting, 2015). The consequence is a multi-organ disease, with the most severe manifestations involving the respiratory and gastrointestinal systems. In the lungs, defective Cl^−^ secretion causes an impairment of mucociliary clearance that favors the bacterial colonization of the airways with airway obstruction, chronic inflammation, and progressive loss of respiratory function (Castellani and Assael, 2017). The lung disease may also arise from defective bicarbonate secretion, which causes loss of innate bactericidal activities and increased viscosity of mucus secretion (Castellani and Assael, 2017).

CFTR is a complex transmembrane protein, consisting of 1,480 amino acids, that includes a cytosolic amino-terminal region, a first membrane spanning domain (MSD1) that is made of six transmembrane helices, a nucleotide binding domain (NBD1), a regulatory (R) domain, a second membrane spanning domain (MSD2) that also includes six transmembrane helices, a second nucleotide binding domain (NBD2), and a cytosolic carboxy-terminal region (Riordan et al., 1989; Csanady et al., 2019; Meng et al., 2019). Opening of the CFTR pore, with the resulting flow of anions, involves the phosphorylation of the R domain by the cAMP-dependent protein kinase A and the binding of two molecules of ATP to the interface between NBD1 and NBD2 (Csanady et al., 2019; Meng et al., 2019). The opening and closing of CFTR pore are regulated by cycles of ATP binding and hydrolysis at the NBDs (Csanady et al., 2019; Meng et al., 2019).

CF-causing mutations are broadly localized along the entire protein sequence. They may cause loss-of-function by a variety of mechanisms [see ref. (De Boeck and Amaral, 2016) for a full description of classes of CF mutations]. The most frequent mutation among CF patients is F508del, with an average frequency of 70% (Beaudet, 1992). This variant, causing the loss of a phenylalanine at position 508 in NBD1, generates a global defect in the folding and stability of CFTR (Veit et al., 2016). Consequently, the mutant protein has a severe trafficking defect consisting of retention in the endoplasmic reticulum and early degradation by ubiquitin-proteasome system. When F508del-CFTR is allowed to traffic to the plasma membrane, by incubation at low temperature or by treatment with pharmacological agents, it displays a “gating defect,” i.e., a longer time spent in the close state (Dalemans et al., 1991; Veit et al., 2016). So far, an effective pharmacological treatment has been developed for patients carrying a single copy or two copies of the F508del mutation (Bacalhau et al., 2023). This treatment includes the combination of two “correctors,” elexacaftor and tezacaftor, acting as pharmacological chaperones on the trafficking defect, with one “potentiator,” ivacaftor, acting on the gating defect (Bacalhau et al., 2023). A previously developed treatment, including the corrector lumacaftor and ivacaftor, had limited efficacy and was only approved for patients with two copies of F508del (Heneghan et al., 2023). So far, structural and biochemical evidence indicates that lumacaftor and tezacaftor, both classified as type 1 correctors bind to a site in MSD1 (Fiedorczuk and Chen, 2022a). Instead, elexacaftor, classified as type 3 corrector, binds to helix 11 of MSD2 and to the “lasso” domain of the amino-terminal region (Fiedorczuk and Chen, 2022b). Other type 1 correctors, not yet available to patients or still under pre-clinical evaluation, are ABBV-2222 [also known as galicaftor (Singh et al., 2020)] and the picomolar potency agent ARN23765 (Pedemonte et al., 2020).

Importantly, pharmacological modulators of CFTR, both correctors and potentiators, have the ability to rescue the function of other CFTR mutants (Bear and Ratjen, 2023; Dreano et al., 2023). This is important for patients carrying rare variants for which there is no available treatment targeting the basic defect.

In the present study, we focused on G85E, a mutation localized in the transmembrane helix-1 of MSD1 and characterized by a severe trafficking defect (Ensinck et al., 2020). According to the data included in the Clinical and Functional Translation of CFTR (CFTR2) database (https://cftr2.org/; accessed on 28 March 2024) the G85E has an allelic frequency of 0.43%. In Italy, however, its allelic frequency is higher, reaching 1.13% (data from the Italian Cystic Fibrosis Registry, 2021-2022). At present, in Europe the G85E variant is still considered orphan of therapies since it is not included among those for which CFTR-modulating drugs have been approved. However, G85E is included in the list of 177 variants for which the triple combination elexacaftor/tezacaftor/ivacaftor has been approved by FDA in the United States.

By using *ex vivo* and *in vitro* airway cell models, we evaluated and compared the efficacy of various type 1 and type 3 correctors, both approved drugs and preclinical compounds, and we identified a combination of molecules that induces a significant rescue of G85E protein function.

## 2 Materials and methods

### 2.1 Patients under study

Six patients compound heterozygous for G85E and a class I CFTR variant (donor IDs: GE004, GE072, FI113, GE143, GE155 and GE227) were included in this study. Donors’ clinical data are shown in Supplementary Material. Two healthy subjects (donor IDs: Ctr032 and Ctr191), one subject homozygous for F508del (donor ID: AN235) and one compound heterozygous for F508del and N1303K (donor ID: AN237) were enrolled as further controls.

### 2.2 Cell culture

Isolation, culture, and differentiation of primary airway epithelial cells were performed as previously reported (Sondo et al., 2022; Terlizzi et al., 2023; Tomati et al., 2023). Briefly, nasal epithelial cells, obtained through a nasal brushing, were cultured and expanded in the serum-free medium PneumaCult Ex-Plus (StemCell Technologies, Vancouver, BC, Canada), supplemented with ROCK and SMAD inhibitors (DMH-1, A-83-01, and Y-27632 compounds). In the first days, the culture medium also contained a mixture of different antibiotics (colistin, piperacillin, and tazobactam) to eradicate bacterial contamination. Differentiated epithelia were obtained by seeding nasal cells on porous membranes (Snapwell inserts, code 3801, Corning Life Sciences, Corning, NY, United States), at high density (500,000 cells/cm^2^). After 24 h, the medium was removed from both sides and replaced with Pneumacult ALI medium (StemCell Technologies, Vancouver, BC, Canada) on the basolateral side only. Epithelia differentiation (up to 16–18 days) was performed in air-liquid interface (ALI) condition. For the short-circuit current analysis, we used well differentiated nasal epithelia with transepithelial resistance (Rt) ranging between 400 and 600 Ω cm^2^.

CFBE41o- cells having stable expression of the halide-sensitive yellow fluorescent protein (HS-YFP) were grown in MEM medium (Euroclone, Milan, Italy) supplemented with 10% FBS, 2 mM L-glutamine, 100 U/mL penicillin, and 100 μg/mL streptomycin (Euroclone, Milan, Italy). For the functional HS-YFP-based assay or CFTR biochemical analysis, CFBE41o- cells were plated at 80% confluence on clear-bottom 96-well black microplates (Corning Life Sciences, Corning, NY, United States) or 12- or 6-wells plates (Euroclone, Milan, Italy).

### 2.3 Chemicals and vectors

The CFTR modulators ivacaftor, tezacaftor, and lumacaftor were purchased from TargetMol (catalog ID: T2588, T2263, and T2595, respectively; Wellesley Hills, MA, United States). Elexacaftor was obtained from MedChemExpress (catalog ID: HY-111772; Monmouth Junction, NJ, United States), ABBV-2222 was from SelleckChem (catalog ID: S8535 Houston, TX, United States), while 4172 was from Life Chemicals (Niagara-on-the-Lake, Canada). ARN23765 was synthesized in house (Pedemonte et al., 2020). CFTR modulators were dissolved in DMSO.

Depending on the type of experiment, the final working concentrations used for the indicated CFTR modulators were as follows: elexacaftor, 3 μM; tezacaftor, 10–30 μM; lumacaftor, 3–10–30 µM; ivacaftor, 1 µM (when applied acutely during short-circuit current measurements or for the YFP assay) or 5 µM (for 24 h treatments). ARN23765 was used at 0.01–0.1–1 μM; ABBV2222 was used at 0.1–1–10 μM; 4172 was used at 10 µM.

Vectors encoding wt-, G85E- and F508del-CFTR variants were purchased from VectorBuilder (vector IDs available upon request; Neu-Isenburg, Germany).

### 2.4 Short-circuit current recordings

Nasal epithelia differentiated on snapwell inserts were mounted in a vertical diffusion chamber resembling a Ussing chamber with internal fluid circulation. Apical and basolateral hemichambers were filled with 5 mL of a solution containing (in mM) 126 NaCl, 0.38 KH_2_PO_4_, 2.13 K_2_HPO_4_, 1 MgSO_4_, 1 CaCl_2_, 24 NaHCO_3_, and 10 glucose. Both sides were continuously bubbled with a gas mixture containing 5% CO_2_–95% air. Measurements were performed at 37°C. The transepithelial voltage was short-circuited with a voltage-clamp (DVC-1000, World Precision Instruments, Sarasota, FL, United States; VCC MC8 Physiologic Instruments, Reno, NV, United States) connected to the apical and basolateral chambers via Ag/AgCl electrodes and agar bridges (1 M KCl in 1% agar). Before the experiment, the transepithelial voltage was clamped at 0 mV after correcting voltage offsets and fluid resistance compensation. The short-circuit current was recorded by analogical to digital conversion on a personal computer.

### 2.5 Transient transfection of CFBE41o- cell line

For the YFP assay, cells were reverse-transfected onto 96-well plates with 0.2 µg per well of the indicated vectors (see “Chemicals and Vectors” Methods Section). To analyse CFTR expression by Western blotting, cells were reverse-transfected onto 12-well plates with 0.8 µg of the indicated vectors, while for the analysis of CFTR half-life cells were reverse-transfected onto onto 6-wells plates with 2 µg of the wt- or G85E-CFTR vectors. Transfection was performed as previously described (Sondo et al., 2022; Terlizzi et al., 2023; Tomati et al., 2023). In brief, cells were transfected in Opti-MEM Reduced Serum Medium (ThermoFisher Scientific, Waltham, MA, United States) using Lipofectamine 2000 (ThermoFisher Scientific, Waltham, MA, United States) as transfection agent. Opti-MEM was carefully replaced, after 6 h, with culture medium without antibiotics. Twenty-four h after transfection and plating, cells were treated with correctors or vehicle alone (DMSO) at the desired concentrations and incubated at 37°C for an additional 24 h, prior to proceeding with the functional HS-YFP-based assay or CFTR biochemical analysis.

### 2.6 YFP-based assay for CFTR activity

CFTR activity was determined by the HS-YFP microfluorimetric assay on CFBE41o-cells as previously described (Sondo et al., 2022; Terlizzi et al., 2023; Tomati et al., 2023). Briefly, prior to the assay, CFBE41o- cells were washed with PBS (137 mM NaCl, 2.7 mM KCl, 8.1 mM Na_2_HPO_4_, 1.5 mM KH_2_PO_4_, 1 mM CaCl_2_, and 0.5 mM MgCl_2_) and then incubated for 25 min with 60 µL per well of PBS containing forskolin (20 µM) and ivacaftor (1 µM), at 37°C, to maximally stimulate the CFTR channel. Cells were then transferred to a microplate reader (FluoStar Galaxy or Fluostar Optima; BMG Labtech, Offenburg, Germany), equipped with high-quality excitation (HQ500/20X: 500 ± 10 nm) and emission (HQ535/30M: 535 ± 15 nm) filters for YFP (Chroma Technology, Bellows Falls, VT, United States). Each assay consisted of a continuous 14-s YFP fluorescence recording with 2 s before and 12 s after injection of 165 µL of an iodide-containing solution (PBS with Cl^−^ replaced by I^−^; final I^−^ concentration 100 mM). After subtracting the background, fluorescence data were normalized to the initial value. The I^−^ influx rate was determined by fitting, for each well, the final 11 s of the data with an exponential function to extrapolate the initial slope (dF/dt).

### 2.7 Analysis of CFTR half-life by western blotting

The day after transfection and plating, CFBE41o- cells were incubated with vehicle alone (DMSO) or with ELX/TEZ (3 µM/10 µM) or ELX/ARN23765 (3 µM/1 µM). Twenty-four hours after treatment with test compounds, CFTR half-life was evaluated by cycloheximide chase. Briefly, CFBE41o- cells were treated with cycloheximide (CHX; 150 μg/mL) (SigmaAldrich, St. Louis, MO, United States) and lysed with RIPA buffer (50 mM Tris-HCl pH 7.4, 150 mM NaCl, 1% Triton X-100, 0.5% Sodium deoxycholate, 0.1% SDS) plus complete proteases inhibitors (Merck KGaA, Darmstadt, Germany) at different time points (0, 3, 6 h) (Tomati et al., 2018). Lysates were separated by centrifugation (15,000 x g at 4°C for 10 min) and supernatant protein concentration was evaluated using the BCA assay (ThermoFisher Scientific, Waltham, MA, United States) following the manufacturer’s recommendation. For each sample, 25 µg of total protein lysates were separated onto a 4%–15% gradient Criterion TGX gel (Bio-rad Laboratories Inc., Hercules, CA, United States) and analyzed by Western blotting (see Analysis of the CFTR expression pattern by Western blotting Methods section).

### 2.8 Analysis of the CFTR expression pattern by western blotting

Lysates of primary nasal epithelia were generated following the previously described protocol (Sondo et al., 2022; Tomati et al., 2023). In brief, to remove the mucus excess, the apical side of HNEC differentiated epithelia (ALI conditions for 16–18 days) were washed with warm HBSS (137.93 mM NaCl, 5.33 mM KCl, 0.338 mM Na_2_HPO_4_, 0.441 mM KH_2_PO_4_, 0.406 mM MgSO_4_, 1.261 mM CaCl_2_, 0.492mM MgCl2, 5.555 mM Glucose) containing 0.4% sodium bicarbonate for 3 h at 37°C. After washing twice with warm complete PBS, the apical side of the filters was dried. Basolateral culture medium (Pneumacult ALI; StemCell Technologies, Vancouver, BC, Canada) was changed to treat cells with indicated correctors or vehicle (DMSO) for 24 h at 37°C. The following day, the newly produced mucus was removed by washing the apical side of epithelia with warm HBSS 0.4% sodium bicarbonate at 37°C for 30 min and then with warm complete PBS. Epithelia were lysed on ice by applying 100 µL/filter of ice-cold RIPA buffer (50 mM Tris-HCl pH 7.4, 150 mM NaCl, 1% Triton X-100, 0.5% Sodium deoxycholate, 0.1% SDS) plus proteases inhibitors (Merck KGaA, Darmstadt, Germany). Cell layers were scraped, collected in a tube, and left on ice for 15 min. To reduce the lysate viscosity, 5 × 22 G needle syringe passages followed by 5 × 27 G needle syringe passages were applied. Lysates were then cleared by centrifugation (15,000× g for 20 min at 4°C). After centrifugation, the supernatant was transferred to a new tube and stored at −80°C for subsequent analysis.

Cell lysates from CFBE41o- cells were generated and then processed as previously described (Sondo et al., 2022; Terlizzi et al., 2023; Tomati et al., 2023). In brief, after transfection (see “Transient Transfection of CFBE41o- Cell Line” Methods Section), CFBE41o- cells were grown to confluence. The day of cell lysis, cells were washed with ice-cold PBS without Ca^2+^/Mg^2+^ and then lysed in RIPA buffer containing a complete protease inhibitor cocktail (Merck KGaA, Darmstadt, Germany). Lysates were separated by centrifugation at 15,000 × g at 4°C for 10 min CFBE41o- or HNE cells supernatant protein concentration was calculated using a BCA assay (ThermoFisher Scientific, Waltham, MA, United States) following the manufacturer’s instructions. Proteins (25 µg for cell lines and 50 µg for HNE cells epithelia) were separated onto gradient 4%–15% Criterion TGX Precast gels (Bio-rad Laboratories Inc., Hercules, CA, United States), transferred to a nitrocellulose membrane with a Trans-Blot Turbo system (Bio-rad Laboratories Inc., Hercules, CA, United States) and analyzed by Western blotting. CFTR and GAPDH were detected using the following primary antibodies: mouse monoclonal anti-CFTR (ab596, J.R. Riordan, University of North Carolina at Chapel Hill, and Cystic Fibrosis Foundation Therapeutics); mouse monoclonal anti-GAPDH (sc-32233; Santa Cruz Biotechnology, Inc.); Horseradish peroxidase (HRP)-conjugated anti-mouse IgG (ab97023; Abcam) was used as secondary antibody. CFBE41o- cell lysates immunoblots were subsequently visualized by chemiluminescence using the SuperSignalWest Femto Substrate (ThermoFisher Scientific, Waltham, MA, United States) and images were acquired with Molecular Imager ChemiDoc XRS System (Bio-rad Laboratories Inc., Hercules, CA, United States). Nasal epithelia-derived immunoblots were visualized by chemiluminescence using the SuperSignalWest Dura Substrate (ThermoFisher Scientific, Waltham, MA, United States) and images were acquired with Alliance Mini HD9 Imager System (Uvitec Ltd. Cambridge). Images were analyzed with ImageJ software (National Institutes of Health, Bethesda). Bands were analyzed as region-of-interest (ROI), normalized against the GAPDH loading control.

### 2.9 Immunofluorescence of transfected CFBE41o- cells

CFBE41o- cells were seeded on µ-Slide 8 well chamber support (Ibidi) at a density of 100,000 cells per well in a total volume of 300 µL of complete MEM medium (supplemented with 10% FBS; 1% penicillin-streptomycin; 1% glutamine). The day after, cells were transfected with a transfection mix containing 0.1 µg of total plasmid DNA encoding for the G85E-CFTR variant and 0.5 µL of Lipofectamine 2000 Transfection Reagent (Invitrogen, cat. No. 11668500). The transfection mix was kept on cells for 24 h. Cells were then treated with the combination of CFTR correctors ARN23765 1 µM plus ELX 5 µM. The day after, 100 μg/mL cycloheximide to inhibit protein synthesis for 0, 3 and 6 h. After treatments, cells were fixed by adding 200 µL of 10% neutral buffered formalin (0501005Q, Bio-Optica) for 5 min at room temperature. After three washings in PBS, cells were permeabilized and blocked by adding a blocking buffer containing saponin as a permeabilizing agent, and BSA (1X PBS; 0,5% BSA; 50 mM NH_4_Cl; 0.02% NaNH_3_; 0.05% saponin) for 30 min at room temperature. After blocking, cells were incubated for 1 h at 4**°**C with 200 μL of primary antibody diluted in the blocking buffer. Rabbit anti-CFTR (CFTR-D6W6L Rabbit mAb #78335, Cell Signaling Technology) at 1:200 and mouse anti-ATP1A1 (mouse mAb Clone:464.6 to alpha 1 Sodium ATPase AMab7671) were used as primary antibodies to detect respectively CFTR and ATP1A1.

Following incubation with primary antibody, cells were rinsed 3 times in PBS and then incubated with 200 μL of a solution of secondary anti-rabbit Alexa Fluor–488 conjugated antibody and anti-mouse Alexa Fluor-546 (Invitrogen) both diluted 1:200 in blocking buffer for 1 h in the dark. After further 3 washes in PBS, Hoechst 33342 1:1,000 was applied for 20 min at RT to stain cell nuclei. Image acquisition and processing were done using ZEISS LSM 700 laser scanning confocal microscope and its own imaging software ZenBlue (Zeiss LSM 700, Oberkochen, Germany).

### 2.10 Statistics

The Kolmogorov–Smirnov test was applied to assess the assumption of normality of data. For normally distributed quantitative variables, when comparing more than two groups, a parametric analysis of variance (ANOVA) followed by a *post hoc* test was used to avoid “multiple comparisons error.” As *post hoc* test we applied the Dunnet test to assess statistical significance of the effect of drug treatments, the Tukey test in the case of combinations of drugs, and Bonferroni when comparing selected pairs of treatment. Normally distributed data are expressed as the mean ± SD and significances are two-sided. Differences were considered statistically significant when *p* < 0.05.

## 3 Results

### 3.1 Evaluation of G85E-CFTR function and its response to modulators in patient-derived nasal epithelial cells

**FIGURE 1:**
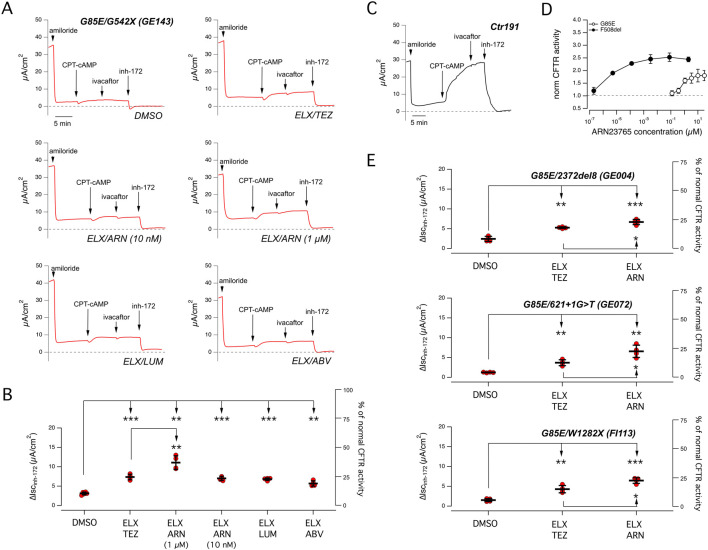
Functional evaluation of CFTR activity and rescue by combined corrector treatment on nasal epithelia derived from CF patients carrying the G85E mutation. **(A)** Representative traces of the effect of vehicle (DMSO), or the combinations of elexacaftor (ELX, 3 µM) with tezacaftor (TEZ, 10 µM), ARN23765 (ARN, 10 nM and 1 µM), lumacaftor (LUM, 3 µM), or ABBV-2222 (ABV, 100 nM) on G85E/G542X nasal epithelial cells (derived from donor ID: GE143) with the short-circuit current technique. During the recordings, the epithelia were sequentially treated (as indicated by downward arrows) with amiloride (10 μM; added on the apical side), CPT-cAMP (100 μM; added on both apical and basolateral sides), ivacaftor (1 μM; apical side) and the CFTR inhibitor-172 (inh-172; 20 μM; apical side). The dashed line indicates zero current level. **(B)** Scatter dot plot showing the summary of results obtained from experiments described in **(A)**. Data reported are the amplitude of the current blocked by 20 μM inh-172 (ΔIsc_inh-172_). For each experimental condition the number of biological replicates was n = 4-6. **(C)** Representative traces recorded with the short-circuit current technique on nasal epithelia derived from a non-CF subject (donor ID: Ctr191) sequentially treated as indicated in **(A)**. **(D)** Dose-response relationships for ARN23765 following 24 h treatment of CFBE41o− cells expressing either F508del or G85E determined with the HS-YFP assay. Each symbol is the mean ± SD of n = 3 experiments. **(E)** Scatter dot plot showing the summary of results obtained from experiments similar to those described in **(A)** following treatment with vehicle (DMSO), with ELX/TEZ (3 µM/10 µM), or ELX/ARN (3 µM/1 µM) on nasal epithelia having genotype G85E/2372del8 (donor ID: GE004), or G85E/621 + 1G>T (donor ID: GE072), or G85E/W1282X (donor ID: FI113). Data reported are the amplitude of the current blocked by 20 μM inh-172 (ΔIsc_inh-172_). For each experimental condition the number of biological replicates was n = 4-6. Symbols indicate statistical significance of treatments: ^∗∗^ *p <* 0.01; ^∗∗∗^ *p <* 0.001.

To study the sensitivity of G85E to pharmacological agents, we adopted a scheme that we previously used in studies on other CF mutations (Capurro et al., 2021; Sondo et al., 2022; Terlizzi et al., 2023; Tomati et al., 2023). First, we tested correctors/potentiators on primary nasal epithelial cells collected from patients with the selected mutation. Then, the results were compared to those obtained by functional and biochemical tests carried out on heterologous expression systems. Figure 1 shows results obtained with nasal epithelial cells from patients carrying the G85E mutation. Given its low frequency, it is nearly impossible to find patients homozygous for this mutation. Accordingly, for our study, we selected patients with G85E in one allele and a mutation in the second allele characterized by negligible CFTR function and null response to correctors and potentiators. In this way, all positive effects of *in vitro* treatment could be unequivocally attributed to G85E-CFTR alone.

Figure 1A shows results obtained with cells from a patient with G85E plus G542X, a nonsense mutation causing severe truncation of CFTR and total loss of function. Differentiated nasal epithelia on porous membranes under air-liquid interface condition were treated for 24 h with different combinations of correctors. In particular, we combined the type 3 corrector elexacaftor (ELX, 3 µM) with different type 1 correctors: tezacaftor (TEZ, 10 µM), ARN23765 (ARN, 10 nM and 1 µM), lumacaftor (LUM, 3 µM), or ABBV-2222 (ABV, 100 nM). After treatment, correctors were removed, and epithelia were mounted in Ussing chamber-like systems for the measurement of CFTR function by short-circuit current recordings. The recordings included the sequential addition of the following agents: amiloride (apical side, 10 µM) to block ENaC-dependent Na^+^ absorption, CPT-cAMP (apical and basolateral, 100 µM) to induce CFTR phosphorylation, ivacaftor (apical, 1 µM) to potentiate CFTR function, CFTR_inh_-172 (apical, 20 µM) to block CFTR. Under control conditions, i.e., epithelia treated with vehicle (DMSO) alone, we found nearly negligible CFTR function. Treatment with a combination of correctors, particularly the combination of ELX plus ARN 1 μM, improved CFTR activity, as indicated by the larger response to CFTR_inh_-172. Figure 1B shows a summary of results of the experiments on differentiated nasal epithelia. The best treatment, ELX plus ARN 1 μM, induced a nearly 3.6-fold increase in CFTR-mediated current, that reached 11.1 ± 0.8 μA/cm^2^ (mean ± SD). Considering that the average amplitude of CFTR function in non-CF epithelia (i.e., the amplitude of the current drop elicited by CFTR_inh_-172) was equal to 28.8 μA/cm^2^ [Figure 1C; similar to the ones previously reported (Terlizzi et al., 2023; Tomati et al., 2023)], the rescued CFTR activity corresponded approximately to 38% of normal CFTR activity. The other treatments were less effective but still statistically significant. The second most effective treatment was ELX plus TEZ, with a nearly 2.4-fold increase in CFTR-mediated current, that reached 7.2 ± 0.3 μA/cm^2^ (mean ± SD), corresponding approximately to 24% of normal CFTR activity. To be noted that ARN has a picomolar potency when tested as corrector of the F508del variant, and it is commonly used at 10 nM (Pedemonte et al., 2020). To understand why ARN was active at 1 µM and not at 10 nM, despite its high nominal potency, we ran dose-response experiments on CFBE41o- cells transfected with G85E- or F508del-CFTR. Cells were treated (24 h) with multiple concentrations of ARN in the picomolar-to-micromolar range. After treatment, CFTR function was determined with the halide-sensitive yellow fluorescent protein (HS-YFP) assay (Pedemonte et al., 2011) which measures the quenching of fluorescence elicited by CFTR-dependent I^−^ influx. Interestingly, we found that the dose-response curve for ARN on the G85E mutant, as compared to F508del, was shifted by several orders of magnitude to higher concentrations, with a maximal effect at 1 µM (Figure 1D).

To confirm the results obtained on the nasal epithelia derived from the first G85E subject, we tested the two best combinations on nasal epithelial cells of other three patients carrying G85E plus one of the following null mutations: 2372del8, 621 + 1G > T, W1282X. Figure 1E shows a summary of results from short-circuit current recordings. The combination ELX plus ARN 1 µM is confirmed as best treatment in the cells of these three additional patients. Accordingly, the best rescue treatment in G85E epithelia corresponds to 25%–35% of normal CFTR function, while ELX plus TEZ rescued G85E function up to 15%–20% of normal CFTR function. To compare the extent of rescue in G85E epithelia, we carried out experiments on cells from F508del/F508del patients (Supplementary Figure S1). In F508del/F508del epithelia, the treatment with ELX plus TEZ generated a CFTR-dependent current of approximately 15 μA/cm^2^, corresponding to 50% of normal CFTR function.

**FIGURE 2:**
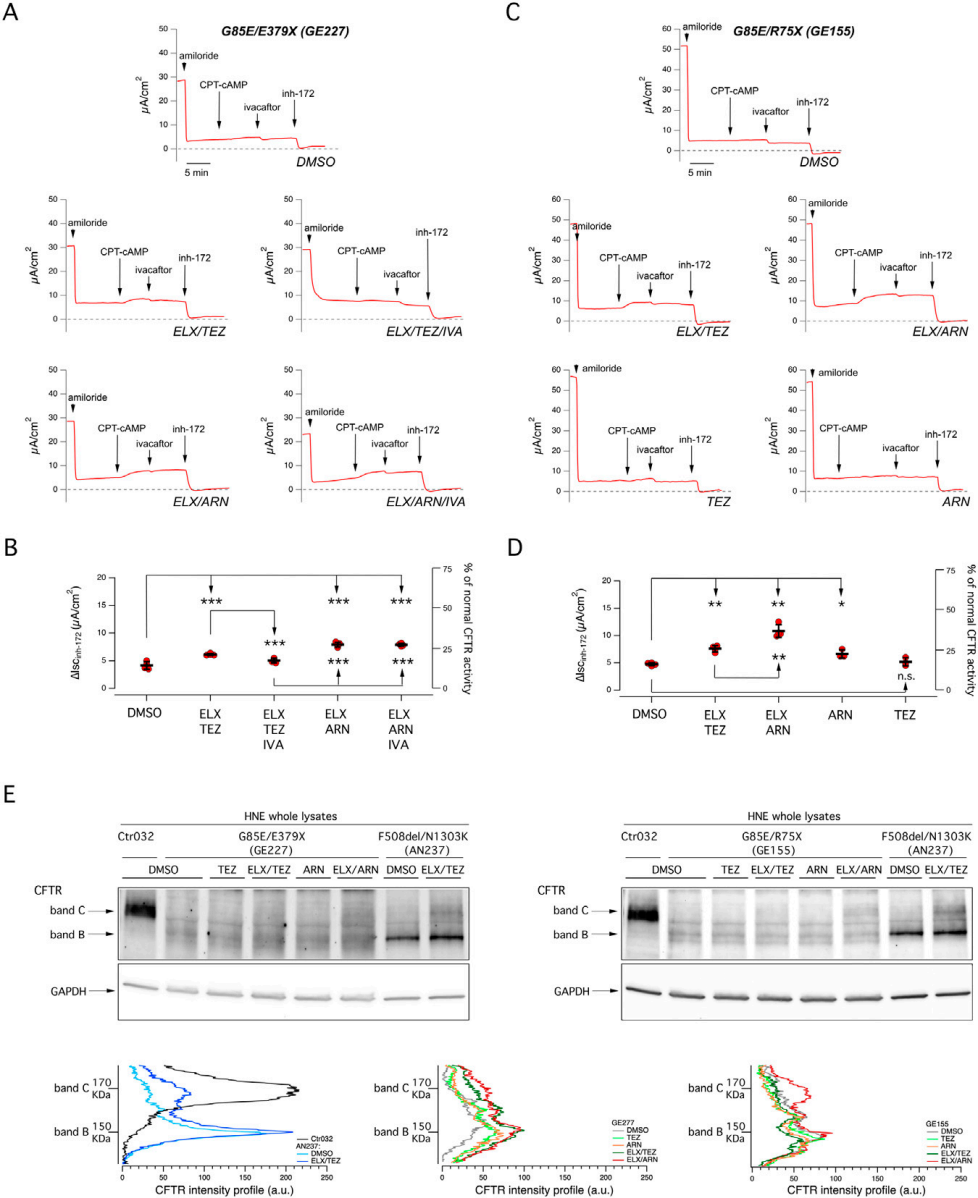
Functional evaluation of the effect of single or combined corrector treatment and of chronic potentiator administration on nasal epithelia derived from CF patients carrying the G85E mutation. **(A)** Representative traces of the effect of vehicle (DMSO), or ELX/TEZ (3 µM/10 µM), or ELX/TEZ/IVA (3 µM/10 µM/5 µM), or ELX/ARN (3 µM/1 µM), or ELX/ARN/IVA (3 µM/1 µM/5 µM) on G85E/E379X nasal epithelial cells (derived from donor ID: GE227) with the short-circuit current technique. During the recordings, the epithelia were sequentially treated (as indicated by downward arrows) with amiloride (10 μM; added on the apical side), CPT-cAMP (100 μM; added on both apical and basolateral sides), ivacaftor (1 μM; apical side) and the CFTR inhibitor-172 (inh-172; 20 μM; apical side). The dashed line indicates zero current level. **(B)** Scatter dot plot showing the summary of results obtained from experiments described in **(A)**. Data reported are the amplitude of the current blocked by 20 μM inh-172 (ΔIsc_inh-172_). For each experimental condition the number of biological replicates was n = 4-6. **(C)** Representative traces of the effect of vehicle (DMSO), or ELX/TEZ (3 µM/10 µM), or ELX/ARN (3 µM/1 µM), or TEZ (10 µM), or ARN (1 µM) on G85E/R75X nasal epithelial cells (donor ID: GE155) with the short-circuit current technique. During the recordings, the epithelia were sequentially treated as indicated in **(A)**. **(D)** Scatter dot plot showing the summary of results obtained from experiments described in **(C)**. Data reported are the amplitude of the current blocked by 20 μM inh-172 (ΔIsc_inh-172_). For each experimental condition the number of biological replicates was n = 4-6. **(E)** Representative Western blot images showing the electrophoretic mobility in lysates of derived from G85E patients (GE227 and GE155) and corresponding density profiles analyses. Epithelia were treated for 24 h with vehicle alone (DMSO) or ELX/TEZ (3 µM/10 µM), or ELX/ARN (3 µM/1 µM), or TEZ (10 µM), or ARN (1 µM) prior to lysis. For comparison, lysates of nasal epithelia derived from one non-CF donor (ID: Ctr032) and one F508del homozygous patient (AN237; treated with DMSO or ELX/TEZ) have been included. Symbols indicate statistical significance of treatments: ^∗^ *p* < 0.05; ^∗∗^ *p <* 0.01; ^∗∗∗^ *p <* 0.001; n. s., not significant.

The pharmacological therapy of CF patients includes the systemic, daily administration of two correctors (ELX/TEZ) and the potentiator ivacaftor. Therefore, to better mimic the situation *in vivo*, we included ivacaftor in the 24 h treatment of epithelia. The rationale of including this experimental condition arises also from reports showing a detrimental effect upon chronic treatment with ivacaftor on the rescue of F508del-CFTR (Cholon et al., 2014; Veit et al., 2014). Moreover, it has been recently reported that acute addition of ivacaftor did not further potentiate cAMP-stimulated chloride secretion in the G85E/G85E epithelia, rather it tended to reduce the transepithelial current (Graeber et al., 2023). Figure 2A and B show the functional data obtained in nasal epithelia of a G85E patient treated with correctors, with/without chronic ivacaftor. A significant decrease in CFTR rescue was observed when ivacaftor was added to the ELX/TEZ combination. Instead, only a negligible, not significant effect was observed when combining ivacaftor with ELX plus ARN 1 µM. We also carried out further experiments to assess the functional effects of TEZ and ARN alone compared to their effects when combined with ELX (Figures 2C, D). We found a significant effect of ARN but not of TEZ as single agents. However, the rescue by ARN alone was significantly smaller than that of the combination with ELX, thus confirming the requirement of the type 3 corrector for maximal effect.

We also investigated this issue by inspecting the electrophoretic mobility of CFTR (Figure 2E). Normal CFTR appears as a 180 kDa band, named band C, that corresponds to the mature fully glycosylated form of the protein. Instead, F508del-CFTR migrates as a 150 kDa band, named band B, that corresponds to the immature core-glycosylated form of the protein retained in the endoplasmic reticulum. Treatment of F508del epithelia with ELX/TEZ combination generates a significant appearance of band C (Figure 2E). Analysis of samples from G85E epithelia confirmed the severe trafficking defect caused by this mutation, with total absence of band C in vehicle-treated condition. In agreement with functional data, the band C mainly appeared with the combination of ELX plus ARN 1 µM (Figure 2E).

### 3.2 Evaluation of G85E-CFTR function and its response to modulators in immortalized bronchial cells

**FIGURE 3:**
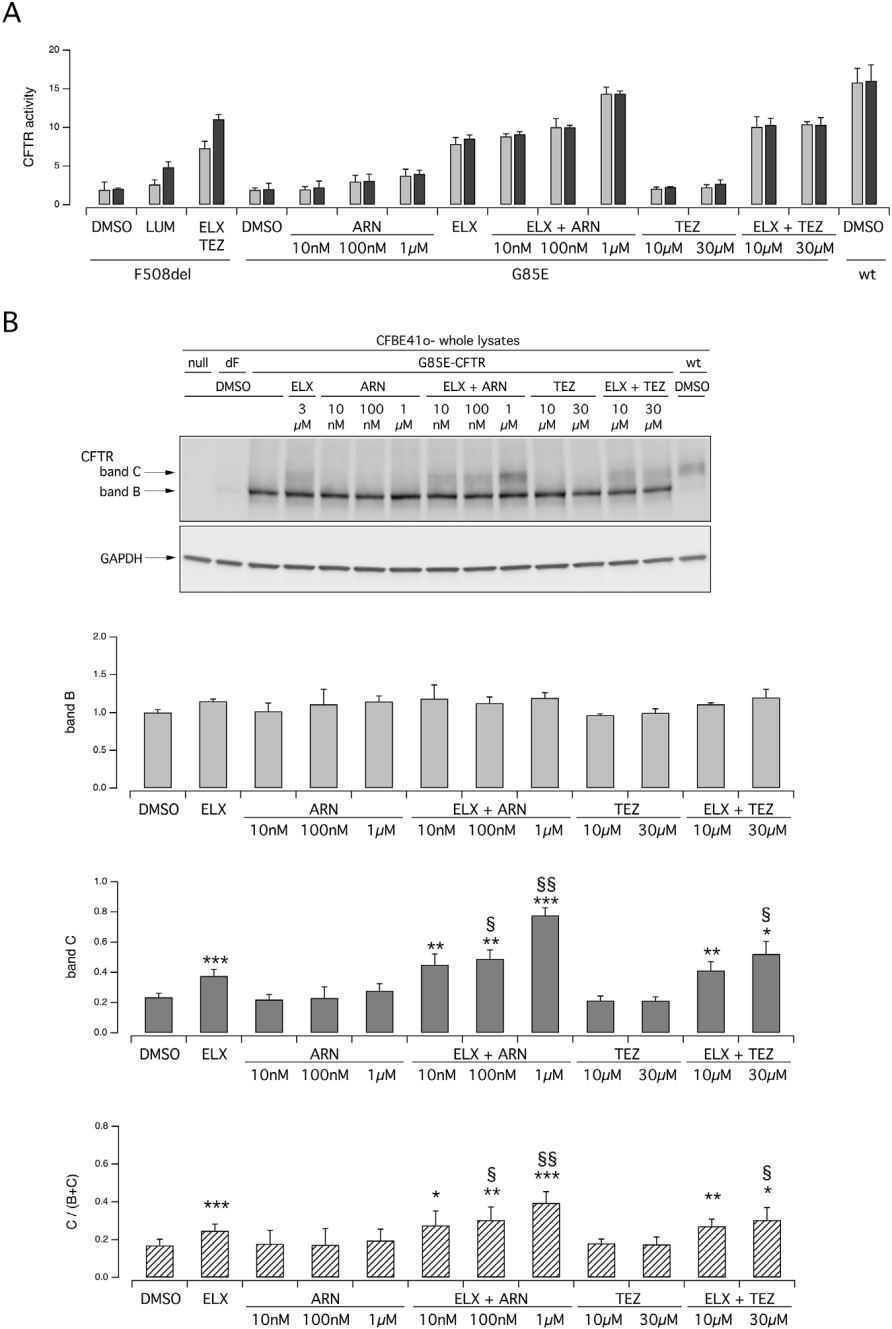
Functional and biochemical evaluation of the effect of tezacaftor and ARN23765, as single agents or combined with elexacaftor, on G85E CFTR mutant in immortalized bronchial cells. The bar graph shows the activity of G85E CFTR transiently expressed in CFBE41o- cells stably expressing the HS-YFP. CFTR activity was determined as a function of the YFP quenching rate following iodide influx elicited by forskolin (20 μM; light gray bars) or forskolin + ivacaftor (1 μM; dark gray bars) in cells treated for 24 h with DMSO (vehicle), or with ARN or TEZ at the indicated concentrations, alone or combined with ELX (3 µM). Data from cells transiently expressing wt-CFTR and F508del- (following treatment with DMSO, or, for F508del only, with LUM (3 µM) or ELX/TEZ (3 µM/10 µM) are also shown for comparison. **(B)** Biochemical analysis of the G85E-CFTR expression pattern in CFBE41o- cells. The representative western blot image shows CFTR electrophoretic mobility in cell lysates following treatment for 24 h, prior to lysis, with the correctors indicated in **(A)**. Lysates from cells transiently expressing wt- and F508del-CFTR are also shown for comparison. Lysates of parental cells have been included as control for antibody specificity. The bar graphs show CFTR band B and band C densitometry, as well as the band C over total CFTR ratio, of the western blot experiments. Symbols indicate statistical significance of treatments: *p < 0.05; **p < 0.01; ***p < 0.001 vs. DMSO-treated; ^§^ *p* < 0.05; ^§§^ *p* < 0.01 vs. ELX-treated.

To further corroborate the results obtained in primary nasal epithelial cells, we carried out experiments on the CFBE41o- bronchial cell line transfected with the expression plasmids coding for wild type, G85E, F508del CFTR (Figure 3). Functional evaluation of CFTR was done with the assay based on HS-YFP (Pedemonte et al., 2011) which measures the quenching of fluorescence elicited by CFTR-dependent I^−^ influx. Figure 3A shows the results obtained for the three versions of CFTR. Results with F508del-CFTR showed the expected behavior with ELX/TEZ treatment being considerably more effective than that with a single (LUM) corrector. Results obtained with G85E essentially confirmed what observed in nasal epithelia. Indeed, the combination of ELX with ARN was the most effective, particularly when ARN was applied at the highest concentration (1 µM). ELX alone was also effective, but less than its combination with ARN. TEZ and ARN were also tested as single agents, but they were not effective (TEZ) or modestly effective (ARN) thus indicating that they require the combination with ELX. Reasoning that, as found for ARN, also other type I correctors might have a dose-response curve shifted to higher concentrations, in these experiments TEZ was used at both 10 and 30 µM. However, no changes in its efficacy as G85E corrector was observed. We also analyzed CFTR maturation in lysates of transfected CFBE41o- cells (Figure 3B). The results were in agreement with functional data. A significant appearance of band C was observed in cells treated with ELX alone. Band C intensity was further increased, by more than two-fold, when ARN 1 µM was included with ELX. Instead, TEZ was less effective compared to ARN in the combination with ELX (Figure 3B).

**FIGURE 4:**
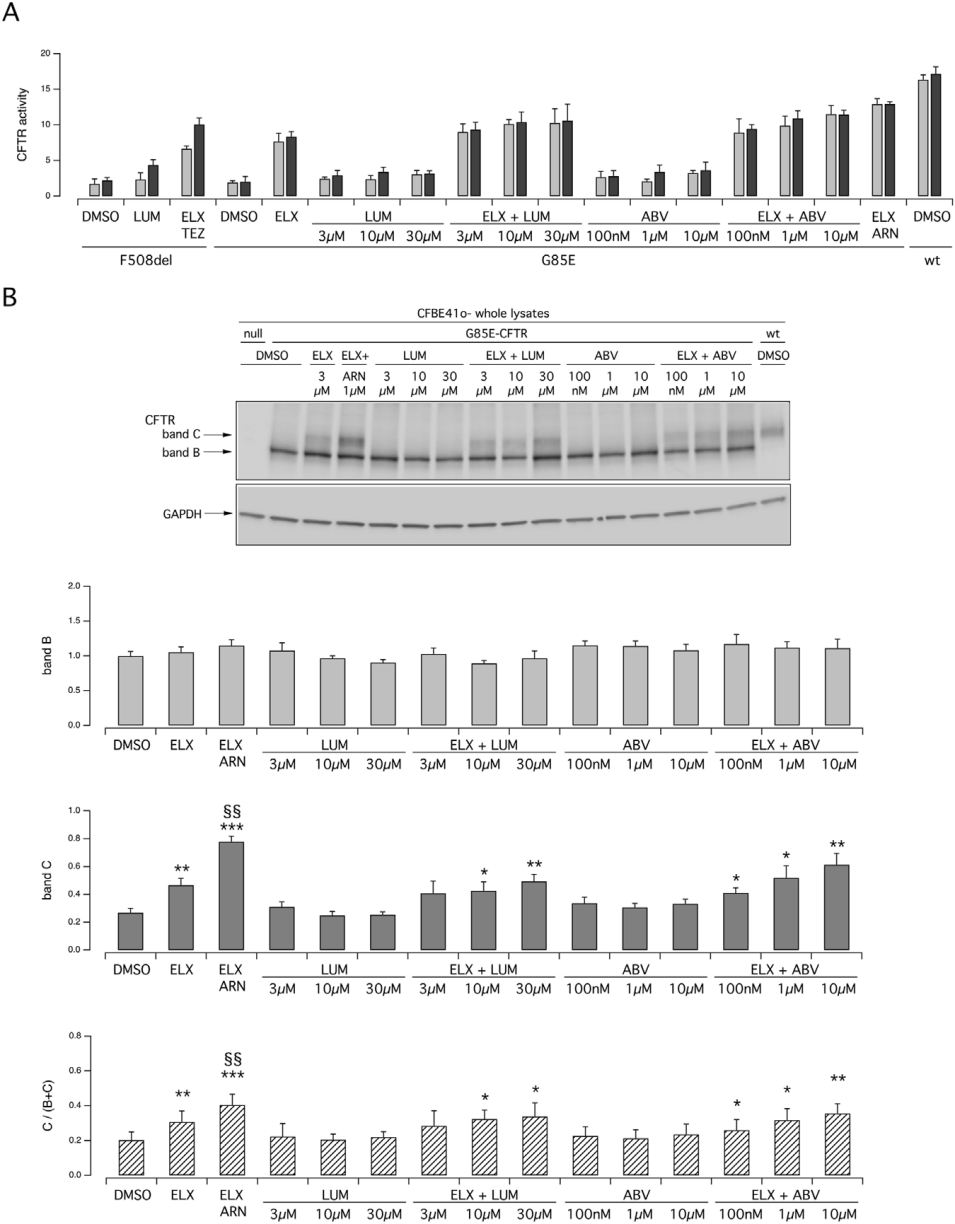
Functional and biochemical evaluation of the effect of lumacaftor and ABBV2222, as single agents or combined with elexacaftor, on G85E CFTR mutant in immortalized bronchial cells. **(A)** The bar graph shows the activity of G85E CFTR transiently expressed in CFBE41o- cells stably expressing the HS-YFP. CFTR activity was determined as a function of the YFP quenching rate following iodide influx elicited by forskolin (20 μM; light gray bars) or forskolin + ivacaftor (1 μM; dark gray bars) in cells treated for 24 h with DMSO (vehicle), or with LUM or ABV at the indicated concentration, alone or combined with ELX (3 µM). Treatment with ELX/ARN (3 µM/1 µM) was included as control. Data from cells transiently expressing wt-CFTR and F508del- (following treatment with DMSO, or, for F508del only, with LUM (3 µM) or ELX/TEZ (3 µM/10 µM) are also shown for comparison. **(B)** Biochemical analysis of the G85E-CFTR expression pattern in CFBE41o- cells. The representative western blot image shows CFTR electrophoretic mobility in cell lysates following treatment for 24 h, prior to lysis, with the correctors indicated in **(A)**. Lysates from cells transiently expressing wt-CFTR are also shown for comparison. Lysates of parental cells have been included as control for antibody specificity. The bar graphs show CFTR band B and band C densitometry, as well as the band C over total CFTR ratio, of the western blot experiments. Symbols indicate statistical significance of treatments: *p < 0.05; **p < 0.01; ***p < 0.001 vs. DMSO-treated; ^§§^ *p* < 0.01 vs. ELX-treated.

We carried out further functional and biochemical experiments on transfected CFBE41o- cells to compare the efficacy of ARN with that of other type 1 correctors, namely, LUM and ABV, at multiple concentrations (Figures 4A, B). Indeed, as done for ARN and TEZ, we tested not only the concentration that are usually used *in vitro*, but also higher concentrations to unmask possibly shifted affinity for the G85E variant. These two agents were also effective when combined with ELX. In the analysis of protein maturation, the combination of ELX/ARN resulted more effective than that of ELX/LUM and ELX/ABV in eliciting the appearance of the band C (Figure 4B).

**FIGURE 5:**
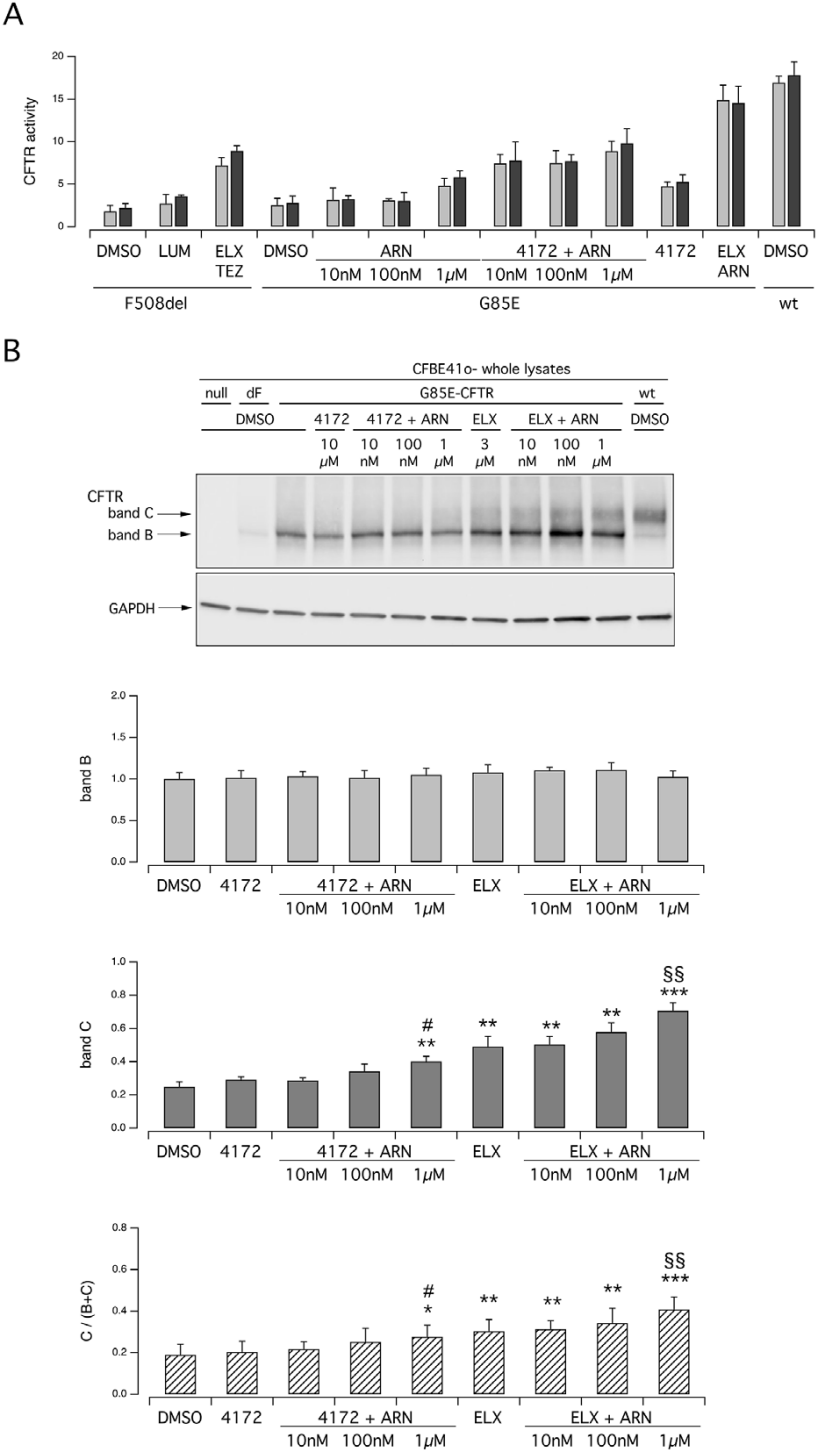
Functional and biochemical evaluation of the effect of ARN23765, as single agent or combined with the type 3 correctors 4172 and ELX, on G85E CFTR mutant in immortalized bronchial cells. **(A)** The bar graph shows the activity of G85E CFTR transiently expressed in CFBE41o- cells stably expressing the HS-YFP. CFTR activity was determined as a function of the YFP quenching rate following iodide influx elicited by forskolin (20 μM; light gray bars) or forskolin + ivacaftor (1 μM; dark gray bars) in cells treated for 24 h with DMSO (vehicle), or with ARN at the indicated concentrations, alone or combined with 4172 (10 µM) or with ELX (3 µM). Data from cells transiently expressing wt-CFTR and F508del- (following treatment with DMSO, or, for F508del only, with LUM (3 µM) or ELX/TEZ (3 µM/10 µM) are also shown for comparison. **(B)** Biochemical analysis of the G85E-CFTR expression pattern in CFBE41o- cells. The representative western blot image shows CFTR electrophoretic mobility in cell lysates following treatment for 24 h, prior to lysis, with the correctors indicated in **(A)**. Lysates from cells transiently expressing wt- and F508del-CFTR are also shown for comparison. Lysates from cells transiently expressing wt-CFTR and F508del- (following treatment with DMSO, or, for F508del only, with ELX/TEZ (3 µM/10 µM) are also shown for comparison. Lysates of parental cells have been included as control for antibody specificity. The bar graphs show CFTR band B and band C densitometry, as well as the band C over total CFTR ratio, of the western blot experiments. Symbols indicate statistical significance of treatments: **p < 0.01; *** *p* < 0.001 vs. DMSO-treated; ^§§^ *p* < 0.01 vs. ELX-treated; ^#^ *p* < 0.05 vs. 4172-treated.

Besides ELX, the compound 4172 has also been classified as a type 3 corrector (Veit et al., 2020). We asked whether 4172 can replace ELX in the combination with ARN. Figure 5A, B show functional and biochemical data respectively. Treatment with 4172 increased the rescue by ARN alone. However, the maximal effect obtained with ARN 1 μM and 4172 10 μM, both at the functional level and in terms of band C intensity, was smaller compared to that of ELX/ARN.

**FIGURE 6:**
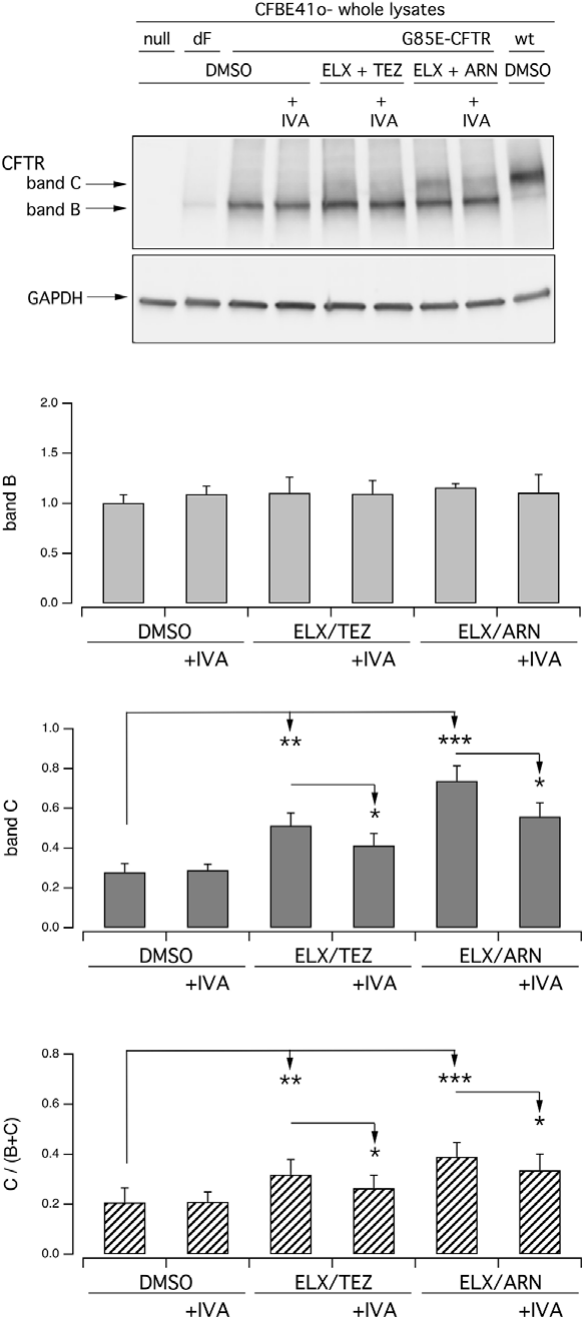
Biochemical evaluation of the effect of chronic ivacaftor on G85E-CFTR rescue by tezacaftor or ARN23765, as single agent or combined with elexacaftor, on G85E CFTR mutant in immortalized bronchial cells. Biochemical analysis of the G85E-CFTR expression pattern in CFBE41o- cells. The representative western blot image shows CFTR electrophoretic mobility in cell lysates following treatment for 24 h, prior to lysis, with vehicle (DMSO), or IVA (5 µM), or ELX/TEZ (3 µM/10 µM), or ELX/TEZ/IVA (3 µM/10 µM/5 µM), or ELX/ARN (3 µM/1 µM), or ELX/ARN/IVA (3 µM/1 µM/5 µM). Lysates from cells transiently expressing wt- and F508del-CFTR are also shown for comparison. Lysates of parental cells have been included as control for antibody specificity. The bar graphs show CFTR band B and band C densitometry, as well as the band C over total CFTR ratio, of the western blot experiments. Asterisks indicate statistical significance of treatments: *p < 0.05; **p < 0.01; ***p < 0.001.

As indicated by functional data on nasal epithelial cells (Figure 2), chronic administration of ivacaftor may decrease the rescue of G85E-CFTR by correctors. We addressed this issue in transfected CFBE41o- cells by analyzing protein maturation. Figure 6 shows that chronic (24 h) ivacaftor significantly decreased the intensity of band C in cells treated together with ELX/TEZ or ELX/ARN compared to correctors alone.

**FIGURE 7:**
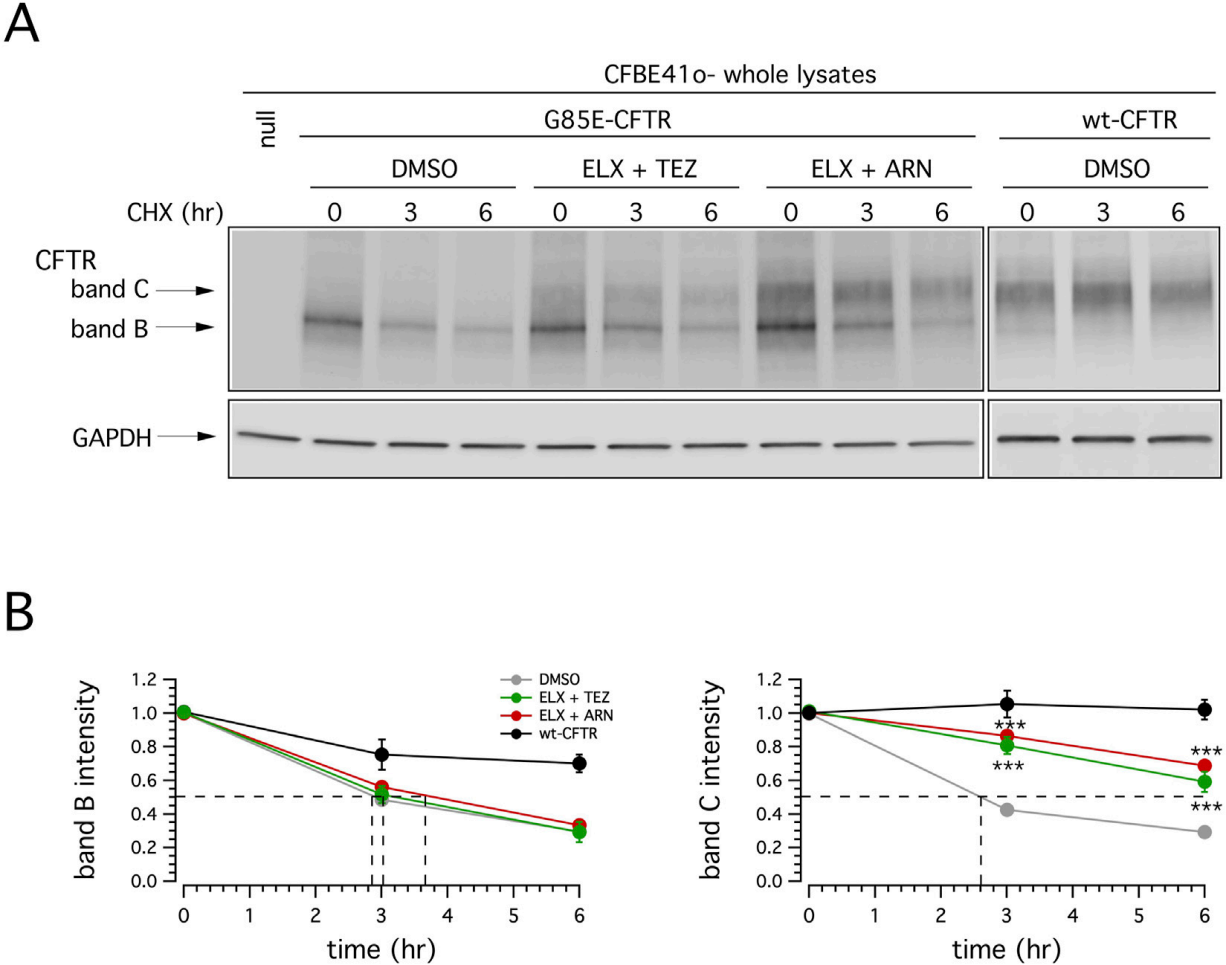
Effect of double correctors treatments on mutant CFTR half-life. **(A)** Immunoblot detection of CFTR in whole lysates derived from wild-type or G85E-CFTR expressing CFBE41o- cells treated with vehicle alone (DMSO), or (for mutant CFTR only) with ELX/TEZ (3 µM/10 µM), or ELX/ARN (3 µM/1 µM), at different time points following CHX-induced block of protein synthesis. For comparison, whole lysates derived from CFBE41o- cells not expressing CFTR (null cells) are also shown as controls for antibody specificity. **(B)** Quantification of wild-type or mutant CFTR (band B and band C) half-life in experiments detailed in **(A)**, normalized by the value at time = 0. Data are means ± SD (n = 3). Dashed lines indicate 50% of the protein remaining (*y*-axis) and the corresponding intercepts on *x*-axis, indicating the estimated half-life.

To further characterize the rescue of mutant CFTR by different correctors combination, we evaluated the degradation rate of G85E CFTR in CFBE41o- cells treated for 24 h with vehicle or ELX/TEZ or ELX/ARN, followed by the block of protein synthesis with cycloheximide (CHX). Cells were then lysed at the initial time point or after 3 or 6 h CHX treatment, and cell lysates were subjected to SDS-PAGE followed by immunoblotting to evaluate mature CFTR expression level. As shown in Figure 7, and consistently with the results reported in Figures 3–5, in vehicle-treated cells G85E-CFTR is mainly expressed as the immature form (band B), and its expression decreases over time. Treatment with ELX/TEZ or ELX/ARN significantly increased the half-life of mature CFTR (band C). The combinations were equally effective on G85E-CFTR stability, with a half-life that exceeded 6 h (Figure 7B). This represents a significant improvement as compared to vehicle-treated cells (∼2.5 h; Figure 7B), although considerably lower than that of wild type CFTR [exceeding 12 h (Heda et al., 2001; Varga et al., 2008)].

**FIGURE 8:**
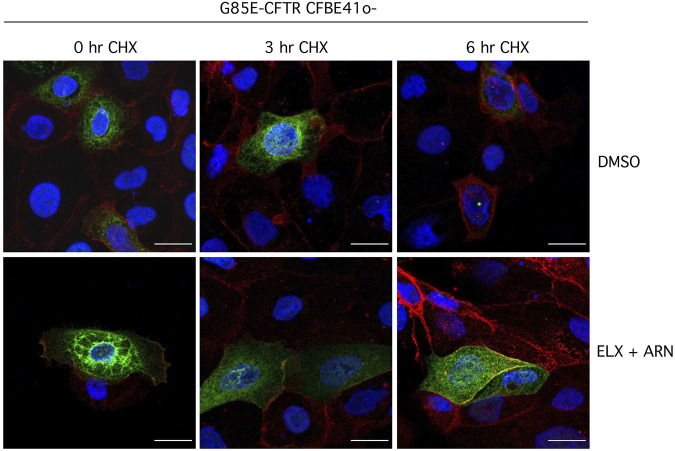
Analysis of CFTR subcellular localization. Representative images showing detection of G85E-CFTR (green) and Na^+^/K^+^-ATPase (red) in CFBE41o- cells by immunofluorescence. Cells were incubated with vehicle alone (DMSO), or ELX/ARN (3 µM/1 µM) for 24 h. Cells were immediately fixed or treated for the indicated time (0–6 h) with CHX and then fixed. Scale bar: 20 µm.

We also investigated the effect of treatment with correctors on the subcellular localization of G85E-CFTR using immunofluorescence. Without treatment, the mutant protein was exclusively present in intracellular compartments (Figure 8). The treatment with ELX/ARN clearly caused the appearance of a peripheral signal, consistent with trafficking of the protein to the plasma membrane. A consistent amount of CFTR protein was however still present in the perinuclear region (Figure 8). Block of protein synthesis with CHX caused the rundown of G85E-CFTR signal. In cells treated with vehicle alone, CFTR signal disappeared after the 6 h CHX treatment. In cells corrected with ELX/ARN, we observed a rapid and marked decrease in G85E-CFTR expression in the perinuclear region, while the membrane-localized protein, overlapping the signal of Na^+^/K^+^-ATPase, was still present after the 6 h CHX treatment (Figure 8).

### 3.3 Efficacy of treatment with modulators on pwCF carrying the G85E variant

During the study, three of the patients described in this work (donor IDs: GE072, G85E/621 + 1G > T; GE143, G85E/G542X, and GE004, G85E/2372del8) started ETI therapy based on their severe clinical condition. Pre-treatment sweat chloride level of GE143 was 68 mmol/L, moved to 70 mmol/L 2 months after starting ETI and to 64 mmol/L two more months later. After 6 months sweat chloride was 42 mmol/L. Other clinical markers were not meaningful, with the exception of an increment in ppFEV1, which raised from 45 to 54. A similar increase in ppFEV1 was observed in GE072, with a shift from 34 pre-treatment to 42 after. In this individual sweat chloride levels decreased from 100 mmol/L to a mean of 62 mmol/L (range 30–91). Before treatment GE004 had 3-4 pulmonary exacerbations per year and sweat chloride of 96 mmol/L; 3 months after starting ETI there have been no exacerbations, weight improved and sweat chloride moved to 57 mmol/L. However, there were no changes in pulmonary function or in oxygen daily needs.

## 4 Discussion

The G85E mutation is characterized by a severe folding defect that causes mutant protein retention in the endoplasmic reticulum and its premature degradation through the proteasomal system, similarly to what observed for the F508del mutation. Interestingly, and differently from the F508del mutation, the G85E-CFTR protein does not display defective channel gating [(Veit et al., 2016); D.N. Sheppard, personal communication]. Thus, increase in intracellular cAMP content, resembling physiological stimulation, is sufficient to activate the mutant channel, once its misfolding has been corrected by means of rescue maneuvers. The G85E mutation is included among those for which the ETI triple combination has been approved by the FDA in the US; however, very few data have been made available on its clinical efficacy so far. In addition, comparative data showing the extent of rescue of G85E or F508del CFTR mutants by ETI in *ex vivo* native cell models are limited.

Our study aimed to evaluate drug responsiveness of the G85E mutation to different CFTR modulators to identify those able to provide an optimal rescue of mutant protein trafficking and function. Our results showed that, in native airway cells, ELX/TEZ rescue G85E-CFTR up to 15%–20% of normal CFTR function. These results are in agreement with a previous study showing that, on rectal organoids, the combination of elexacaftor with the type I correctors lumacaftor or tezacaftor provided only limited benefit in the rescue of CFTR activity, as measured by organoid swelling and plasma membrane density of the protein (Ensinck et al., 2022). Thus, the extent of rescue by treatment with ELX/TEZ is by far smaller than that observed for the F508del, which ranges between 40% and 65% of normal CFTR function, as previously shown by several groups, including ours (Veit et al., 2020; Capurro et al., 2021; Laselva et al., 2021; Sondo et al., 2022). This limited rescue of the folding and trafficking defect appeared to be further decreased by the chronic treatment with IVA. Indeed, it has been demonstrated that prolonged exposure to potentiators, in particular to IVA, can exert a detrimental effect on the rescue of F508del-CFTR by correctors (Cholon et al., 2014; Veit et al., 2014). In keeping with these results, a recent work by the Bear group showed that IVA increased the fluidity of and reorganized the plasma membrane (Chin et al., 2018), supporting a potential for nonspecific effects of IVA on the lipid bilayer that could account for its destabilizing effect on rescued F508del-CFTR (Chin et al., 2018). The authors also demonstrated that IVA exerted a similar negative effect on the stability of other membrane localized solute carriers (SLC26A3, SLC26A9, and SLC6A14), indicating that this negative effect is not specific for F508del-CFTR (Chin et al., 2018). Thus, it is not unexpected that this effect can be seen also with other misfolded CFTR mutants, such as G85E. This is of high relevance because it may further decrease the already limited rescue induced by ELX/TEZ. In addition, since the G85E-CFTR protein does not display a gating defect, as previously reported, there could be no need for the use of a potentiator. On the other side, the inclusion of a potentiator may guarantee maximal activation of the CFTR protein even in the presence of sub-maximal physiological stimulation levels.

Rescue of mutant G85E-CFTR can be increased by treatment with specific corrector combinations. In particular, the most effective combination, ELX/ARN, provided a significantly higher rescue than other combinations, corresponding to 25%–35% of normal CFTR function. Interestingly, in primary nasal epithelia, the detrimental effect of chronic IVA on G85E rescue by ELX/ARN is negligible, while it can be appreciated on immortalized CFBE41o- cells transfected with G85E. The efficacy of G85E rescue appears to be related to the type 1 corrector that is used. The G85E mutation is located in the transmembrane helix-1 of MSD1, thus very close to the binding site of type 1 correctors identified in MSD1 (Fiedorczuk and Chen, 2022a). Our previous study reporting the identification and characterization of ARN as a F508del CFTR corrector suggested that ARN is a type 1 corrector (Pedemonte et al., 2020). Interestingly, our results show that the dose-response relationship of type 1 correctors is shifted to higher concentrations when tested on G85E, as compared to F508del. We can hypothesize that the G85E mutation may alter the binding site of type 1 correctors, possibly decreasing their affinity for the target. ARN is the most potent corrector identified so far, with a EC50 equal to 38 pM and a maximal effective concentration in the 1–10 nM range when tested on the F508del (Pedemonte et al., 2020). However, its dose-response relationship is dramatically shifted towards higher values for the G85E, with a maximal effective concentration at 1 µM. Further studies are however needed to understand whether the G85E mutation has an impact on the binding site of type 1 correctors and its possible consequences in terms of drug potency and efficacy.

The benefit of treatment in the three individuals taking ETI was expressed by decrease of sweat chloride and, in two of them, better pulmonary function. Either improved less than F508del heterozygotes treated with the same compound (Middleton et al., 2019), which appears to be consistent with the *ex vivo*/*in vitro* findings of this paper. Such modest effects obtained by ETI *in vivo* underscore the need to develop more effective compound combinations for patients with difficult-to-treat mutations. In the specific case of G85E, such combinations should include more effective class 1 correctors, like ARN23765, in order to maximize the rescue of the mutant protein.

## Data Availability

The original contributions presented in the study are included in the article/Supplementary Material, further inquiries can be directed to the corresponding author.
