# Supplementary material for: Pharmacological rescue of the G85E CFTR variant by preclinical and approved modulators
Source: Front Pharmacol. 2024 Nov 18;15:1494327. doi: 10.3389/fphar.2024.1494327 (PMC11608983; doi:10.3389/fphar.2024.1494327)
Supplement: Supplementary file 1 [file Image1.PDF]

frontiers

Supplementary Material

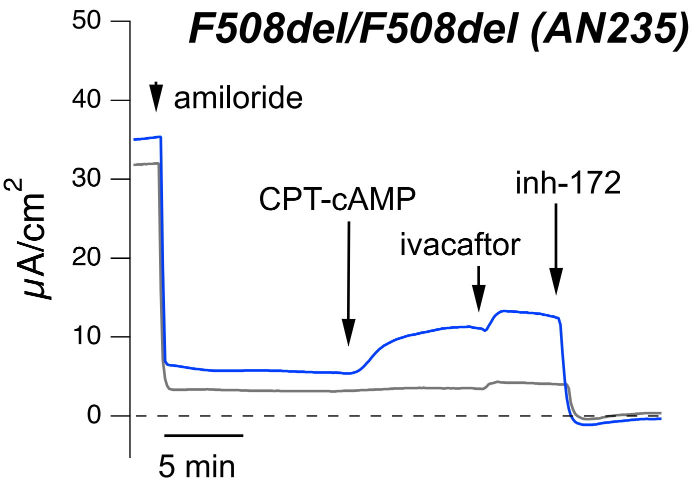

Supplementary Figure 1. Representative traces recorded with the short-circuit current technique on F508del/F508del nasal epithelia (derived from donor ID: AN235) pre-incubated for 24 hr with vehicle (DMSO; gray trace), or ELX/TEZ (3 µM / 10 µM; blue trace) and sequentially treated (as indicated by downward arrows) with amiloride (10 µM; added on the apical side), CPT-cAMP (100 µM; added on both apical and basolateral sides), ivacaftor (1 µM; apical side) and the CFTR inhibitor-172 (inh-172; 20 µM; apical side). The dashed line indicates zero current level.
